# Technology Innovation for Discovering Renal Autoantibodies in Autoimmune Conditions

**DOI:** 10.3390/ijms252312659

**Published:** 2024-11-25

**Authors:** Maurizio Bruschi, Giovanni Candiano, Andrea Petretto, Andrea Angeletti, Pier Luigi Meroni, Marco Prunotto, Gian Marco Ghiggeri

**Affiliations:** 1 Laboratory of Molecular Nephrology, IRCCS Istituto Giannina Gaslini, Via Gaslini, 16147 Genova, Italy; mauriziobruschi@gaslini.org (M.B.); giovannicandiano@gaslini.org (G.C.); andreaangeletti@gaslini.org (A.A.); 2 Department of Experimental Medicine (DIMES), University of Genoa, 16132 Genova, Italy; 3 Core Facilities-Proteomics Laboratory, IRCCS Istituto Giannina Gaslini, 16147 Genova, Italy; andreapetretto@gaslini.org; 4 Experimental Laboratory of Immunological and Rheumatologic Researches, Istituto Auxologico Italiano–Istituto di Ricovero e Cura a Carattere Scientifico, 20145 Milano, Italy; pierluigi.meroni@unimi.it; 5 Institute of Pharmaceutical Sciences of Western Switzerland, University of Geneva, 1205 Geneva, Switzerland

**Keywords:** technology evolution, immunopeptidomics, immunoproteomics, STED, autoantibodies, autoimmune diseases

## Abstract

Autoimmune glomerulonephritis is a homogeneous area of renal pathology with clinical relevance in terms of its numerical impact and difficulties in its treatment. Systemic lupus erythematosus/lupus nephritis and membranous nephropathy are the two most frequent autoimmune conditions with clinical relevance. They are characterized by glomerular deposition of circulating autoantibodies that recognize glomerular antigens. Technologies for studying renal tissue and circulating antibodies have evolved over the years and have culminated with the direct analysis of antigen–antibody complexes in renal bioptic fragments. Initial studies utilized renal microdissection to obtain glomerular tissue. Obtaining immunoprecipitates after partial proteolysis of renal tissue is a recent evolution that eliminates the need for tissue microdissection. New technologies based on ‘super-resolution microscopy’ have added the possibility of a direct analysis of the interaction between circulating autoantibodies and their target antigens in glomeruli. Peptide and protein arrays represent the new frontier for identifying new autoantibodies in circulation. Peptide arrays consist of 7.5 million aligned peptides with 16 amino acids each, which cover the whole human proteome; protein arrays utilize, instead, a chip containing structured proteins, with 26.000 overall. An example of the application of the peptide array is the discovery in membranous nephropathy of many new circulating autoantibodies including formin-like-1, a protein of podosomes that is implicated in macrophage movements. Studies that utilize protein arrays are now in progress and will soon be published. The contribution of new technologies is expected to be relevant for extending our knowledge of the mechanisms involved in the pathogenesis of several autoimmune conditions. They may also add significant tools in clinical settings and modify the therapeutic handling of conditions that are not considered to be autoimmune.

## 1. Introduction

Autoimmune glomerulonephritis represents a homogeneous area of renal pathology with clinical relevance in terms of its numerical impact and difficulties in its treatment. It is characterized by glomerular deposition of circulating autoantibodies that recognize renal antigens and usually take part in a more generalized autoimmune process involving other organs such as in systemic lupus erythematosus (SLE)/lupus nephritis (LN), ANCA-related vasculitis, and Goodpasture syndrome. Only a few autoimmune forms have the kidney as a unique target (i.e., membranous nephropathy—MN). The pre-requisite for defining a circulating autoantibody as the cause of glomerulonephritis is the demonstration that it is deposited in mesangial areas or along the basement glomerular membrane with subendothelial or subepithelial localization. A second step is to study the cell origin of each antibody, which is crucial when defining therapies.

Technologies for studying tissue and circulating antibodies have evolved over the years and culminated with the analysis of the antigen–antibody complex in renal bioptic fragments. The combination of tissue microdissection of glomeruli and mass spectrometry (MS) has played a crucial part in the evolution from indirect techniques based on immunofluorescence to direct tissue analysis. Tissue microdissection is carried out on kidney fragments deriving from needle biopsies performed for diagnostic purposes from patients with urinary alterations. The tissue is first dissected under microscopy guide to obtain glomeruli free of any other renal segments, and homogenates of isolated glomeruli are then separated with two-dimensional electrophoresis; in the final part, those proteins recognized by serum under Western blot are characterized by MS.

Direct MS of immunoprecipitates of either glomerular and renal homogenates deriving from renal biopsies is the most recent methodologic evolution that, in the case of total renal homogenates, eliminates the need for tissue microdissection. Direct MS is based on the assumption that, in autoimmune conditions, immunoprecipitated autoantibodies are part of the disease, so this technique requires further validation by microscopy techniques and ELISA. For its simplicity, direct immunoprecipitation followed by MS is now considered the technique of choice for clinical applications. The results obtained with mass spectrometry of tissue microprecipitates have led to the discovery of several new glomerular antigens in MN and LN that are now utilized for the subtypes of both pathologies.

Peptide and protein arrays for the characterization of circulating autoantibodies are new high-throughput technologies. Both are very sensitive techniques which allow the characterization of hundreds of new antibodies in circulation. The contribution of arrays to amplify the panel of potential autoantibodies involved in glomerulonephritis is expected to be relevant for improving our knowledge of the pathogenesis of several autoimmune conditions. Because of their high cost, the arrays have so far only been utilized in basic research studies.

## 2. Technology Innovation for Renal Autoantibody Discovery

### 2.1. Indirect Approaches

The identification of autoantibodies and glomerular antigens in renal autoimmune pathologies started almost 50 years ago with the characterization of Goodpasture syndrome. At that time, laser dissection technology of glomeruli necessary for the direct microelution of antibodies was not available, and studies utilized an indirect approach in which circulating autoantibodies were challenged with homogenates of normal kidneys and later with human podocytes maintained ‘in culture’ based on the consideration that these cells are the functional drivers of the permeability properties of the kidney [1,2]. The protein sequence of spots recognized in the sera of patients after monodimensional electrophoresis has been, for a few decades, the technique utilized for their characterization. The discovery of glomerular basement membrane (GBM) antigens in Goodpasture syndrome was the first important result deriving from this approach that posed the basis for a successive analysis of relevant epitopes involved in the pathogenesis of the disease. Goodpasture syndrome remained for years an isolated example of a study addressing renal targets of an autoimmune process. Until the development of two-dimensional electrophoresis, the major drawback of this approach was the poor quality of separation of protein mixtures; with two-dimensional electrophoresis and, more importantly, mass spectrometry began a new phase of the research on renal autoantibodies that allowed, between 2010 and 2020, the discovery of several autoantibodies involved in MN and LN (see below).

### 2.2. Laser Microdissection and Characterization of Microeluates

**Figure 1:**
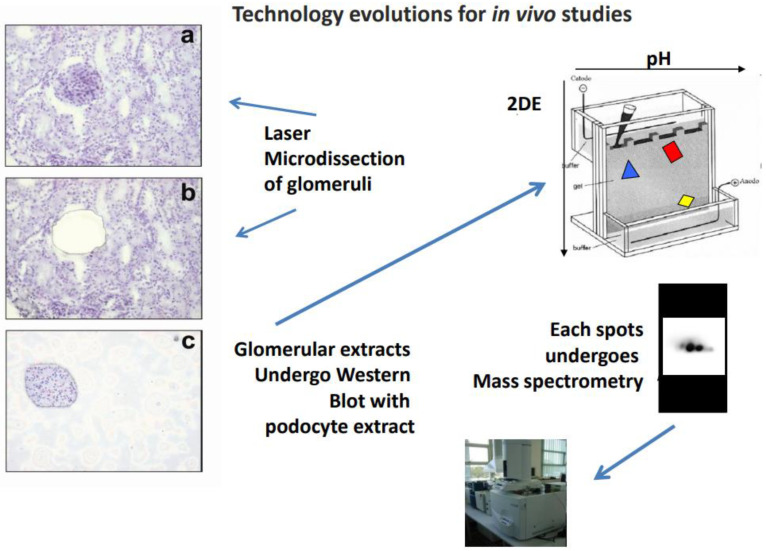
Workflow utilized for characterizing glomerular antibodies microeluted from the kidney. Glomerular microdissection is the first step: (**a**,**b**) show a renal bioptic sample before and after microdissection, and (**c**) shows the glomerulus derived from the procedure. Glomerular extracts are then incubated with podocyte proteins previously separated by 2D electrophoresis and transferred to nitrocellulose membranes. Those spots that are recognized by immunoglobulin glomerular extracts undergo characterization by mass spectrometry.

**Figure 2:**
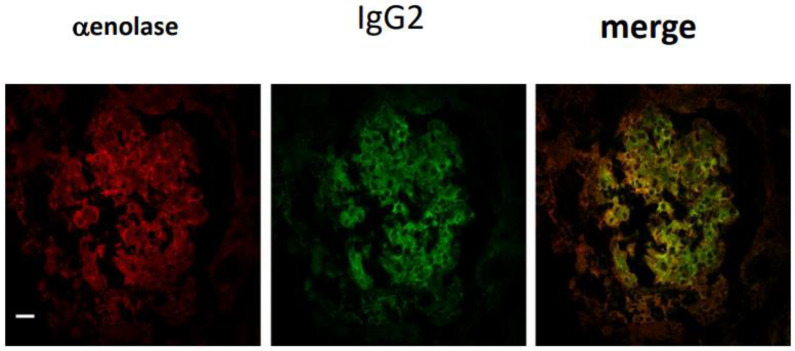
Validation of antibodies microeluted from glomeruli and characterized by immunoblot and mass spectrometry is carried out by immunofluorescence on kidney biopsies. The example presented in this figure is the validation of alpha-enolase as an antigen in patients with lupus nephritis. In this case, alpha-enolase, stained in red, and IgG2, stained in green, have an intense yellow merge, which indicates that the two proteins interact in the tissue. Magnification: ×400.

Membranous nephropathy and lupus nephritis are two autoimmune conditions characterized by the presence of antibodies in mesangial and subepithelial granular deposits that differ for their isotype, i.e., IgG4 in MN, and IgG2 in LN. The identity of antigen targets of autoimmunity was unknown (or hypothetical) for years until laser microdissection of glomeruli from renal biopsies of patients was made available. In this way, antibodies were microeluted from dissected glomeruli in vivo and exposed to homogenates of normal glomerular proteins separated with 2D electrophoresis. Glomerular proteins recognized by microeluted antibodies in Western blot were then identified by MS (Figure 1) [1,2,3,4,5]. Confirmation of the interaction between microeluted antibodies and identified antigens was given by co-staining the characterized antigen with an immunoglobulin in granular subepithelial deposits or in mesangial areas (Figure 2). In almost all autoimmune glomerulonephritis cases, the amount of immunoglobulins deposited in glomeruli, in general, IgG, is sufficient to be detected by common fluorescence microscopies. The availability of microscopes defined as ‘super-resolution’ (STED) has demonstrated that limited deposits of IgG in the slit diaphragm may occur in conditions that have been considered nonautoimmune for years, such as minimal change disease (MCD) and focal segmental glomerulosclerosis (FGS). This new finding has determined a re-definition of MCD and FGS on a critical basis (see below). Finally, based on results deriving from microdissection-mass spectrometry, specific ELISAs (Figure 1) were developed for a definitive confirmation of the presence of specific autoantibodies in the circulation and glomeruli of MN and LN patients. The results have completely modified the interpretation of the events leading to MN and LN (see the application Section 4).

### 2.3. Limited Proteolysis of Tissue

This technique represents an evolution from the classical proteomic approach of isolated glomeruli since immunoprecipitation has been substituted in place of laser microdissection. The material deriving from limited proteolysis of a kidney bioptic sample obtained for clinical purposes is first immunoprecipitated with Protein A or G and the immunoprecipitate is analyzed by mass spectrometry. The rationale of this approach is that in any glomerular autoimmune condition, immunocomplexes that can be immunoprecipitated after limited proteolysis from a renal bioptic fragment are derived from glomeruli, and the antigen characterized in immunoprecipitates is the antigen responsible for the autoimmune reaction. The method includes an initial preparation of the tissue during which bioptic renal fragments are incubated with a mixture of proteolytic enzymes and the homogenate is then mechanically disrupted; partial digests are then centrifugated and immunoprecipitated with Protein A or G Dynabeads. The material that is magnetically immobilized is then analyzed by mass spectrometry [6,7]. In just a few years, 10 new antigens have been discovered in MN and LN, representing an important evolution in the area of glomerular autoimmunity [7,8,9,10,11,12,13,14,15,16]. Details are given in the dedicated section.

### 2.4. Magnetic Beads’ Immunoprecipitation for ‘Highly Reactive Antibodies’

The use of enzyme-linked immunosorbent assays (ELISAs) in clinical chemistry has been consolidated over the years. In the case of antigens with complex structures or repeated domains, ELISAs may produce unclear results; prior precipitation of circulating antibodies with Protein A/G followed by either ELISA or Western blot is a valid alternative to avoid bias. One example is nephrin, which presents in its sequence six immunoglobulin-like repeats and one fibronectin-like domain that characterize other proteins and may lead to false positives. An unbiased method for anti-nephrin antibody determination includes a first step in which recombinant nephrin tagged with poly-histidine (from 6 to 8 poly-histidine amino acids), Flag (DYKDDDDK amino acids), or other tags (glutathione *S*-transferase, maltose-binding protein, thioredoxin, twin strep-tag, etc.) is added to serum and allowed to bind circulating anti-nephrin IgG. The complex is then immunoprecipitated by Protein A/G immobilized to a solid support such as magnetic beads or agarose resin. At the end of the incubation step, the nephrin–IgG2 immunocomplexes are pelleted by a magnet (or by centrifugation in case of other supports) and eluted using an acidic solution (pH 2.5). The use of magnetic beads in place of agarose has a few advantages since, besides their easy removal, magnetic beads minimize sample loss and reduce possible mechanical shearing of fragile biomolecules. They are also more compatible with automated systems for high-throughput applications.

Quantitation of nephrin in the immunoprecipitate may be carried out with a Western blot that excludes any potential substance that interacts with the His tag from the analysis. Quantitation of Flag-tagged nephrin does not require Western blot because this tag does not bind contaminants; in this case, nephrin can be quantified after immunoprecipitation by ELISA.

### 2.5. High-Resolution Microscopy

Stimulation emission depletion microscopy (STED) is a fluorescence-based technique developed in 1994 [17] and was first applied in biology in the 21st century [18]. It creates super-resolution images up to 70 nm with a sensitivity resembling the electron microscopy approach. STED exploits the nonlinear response of fluorophores, improving the resolution of the antigen–antibody complexes, and produces high-quality images, providing a clear demonstration of their co-localization. Concerning glomerular pathologies, STED can resolve the fine structures of the slit diaphragm with sensitivity at the nanometer scale.

The application of STED to human research has only been initiated recently and is still limited by costs [19,20]. In autoimmune pathologies of the kidney, STED application is of fundamental importance in showing limited antibody deposition that is not resolved by common optical techniques. MCD and FGS are the areas of application of STED in basic research with potential immediate clinical application. The demonstration of the co-localization of IgG with nephrin in the slit diaphragm using STED has completely changed the interpretation of ‘orphan’ renal diseases such as idiopathic nephrotic syndrome that have previously been considered to be of uncertain origin but can now be considered autoimmune diseases (anti-nephrin antibodies) [17].

### 2.6. Peptide Arrays

Peptide and protein arrays have been recently utilized for discovering new circulating autoantibodies. The peptide array consists of 7,499,126 peptides with 16 amino acids each that together cover the amino acid sequence of all the proteins coded by the human genome. Each peptide has the same sequence as the consecutive peptide except for three amino acids at the tail, a model that is replicated infinite times to obtain a multiple of the three different amino acids. Considering that to identify a protein, the minimum amount of amino acids in a sequence is 54, an informative sequence is formed by the starting peptide (16 amino acids) plus the 3 amino acids at the tail of two contiguous peptides of 13 consecutive peptides whose sum is 39 amino acids. In fact, the concept of the peptide array is that to identify an antigen, an antibody should react with 14 consecutive peptides. Based on these considerations, it appears that the array covers the amino acid sequence of 535,651 proteins, a number obtained by dividing the 7,499,126 peptides of the array by 14. The basic procedure is simple and consists of incubating the sera of patients with all 7,499,126 peptides from the customized array, and the intensity of the relative fluorescence deriving from their interaction is aligned in sequence by informatic technologies to obtain the 14 peptides necessary for the identification of a protein. This statistical approach requires specific techniques of analysis aimed at obtaining a probabilistic identification of epitopes known as a ‘sliding window’ (S-PIE) that is based on the concept that for true epitopes, a signal above a given threshold should be detectable for all consecutive peptides containing the recognized amino acid sequence. Given the probability (P) that a peptide with a fluorescence intensity higher than the threshold (I_0-x_) is a false positive, the probability of two consecutive peptides being false positive is equal to P2. The same holds for longer stretches of peptides P3, P4, P5……, the sum of which enables the identification of an epitope with a very high probabilistic score of P14 for the stretch of 14 peptides required for the identification of a protein. In general, several proteins are identified with the peptide array and require a second statistical step to evaluate the effective significance in comparison with either normal sera or other diseases. Analyses based on multiple samples simplify the search for significance. One is the weight gene co-expression network analysis immuno-intensity data (W-GCNA) that are used to remove all peptides with a mean intensity near the normal range, and the other is Volcano plots and hierarchical cluster analysis with heat maps.

**Figure 3:**
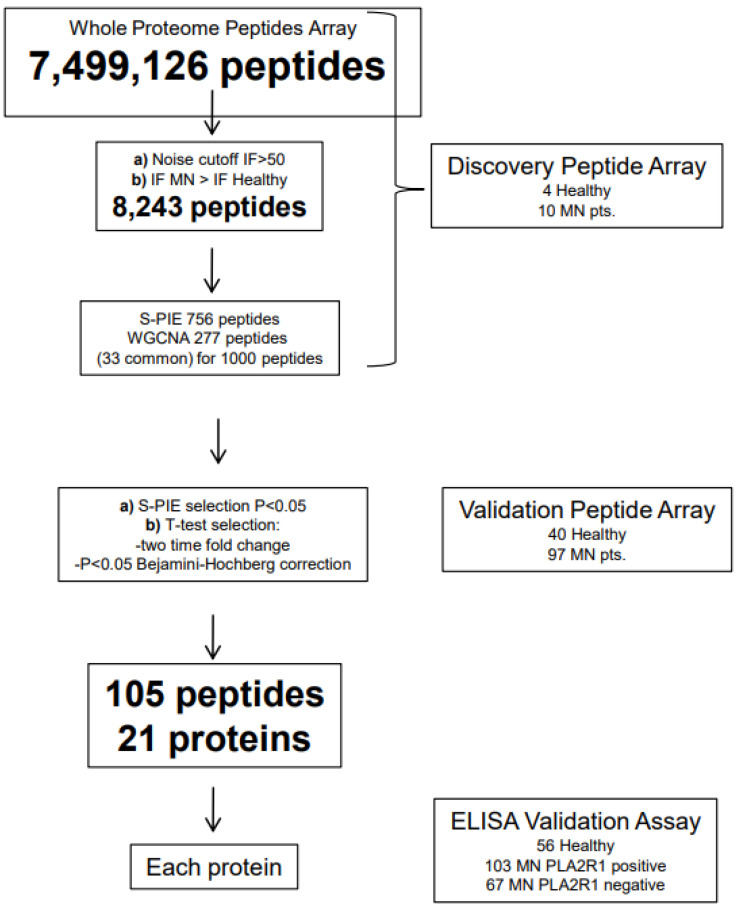
Whole proteome peptide arrays consist of 7,499,126 peptides with 16 amino acids each that together cover the amino acid sequence of all the proteins coded by the human genome. Sera are incubated with all 7,499,126 peptides of the customized array, and the intensity of the relative fluorescence deriving from their interaction is aligned in sequence by informatic technologies to obtain the identification of a unique linear epitope corresponding to a specific protein. This figure shows the application of the peptide arrays for discovering new circulating antibodies in patients with membranous nephropathy, which is a unique example of the application of the array in human pathology [17].

The peptide array has so far been utilized to discover new circulating autoantibodies in MN only [18] but other studies on other glomerulonephritis are currently in progress (Figure 3). Many proteins representing potential new antigens were identified in MN and, among them, formin-like-1 (FMNL1), a protein endowed in the podosomes of macrophages, emerged as the most relevant.

One possibility to reduce the complexity of the procedure (and the cost) is to limit the number of peptides to a reduced number of proteins such as those implicated in specific pathologies. One example is LN, in which case, an array would contain proteins of immunologic interest. The customized array designed for LN contains less than 1000 proteins, which means there are 54,000 peptides. Considering the 537,661 proteins of the original array, the 1000 proteins of the personalized array represent a notable reduction that impacts costs in a significant way.

An important characteristic of the peptide array is the definition of the epitope recognized by a specific antibody, an element that could have relevance for designing specific ELISAs (see below).

### 2.7. Protein Array

This array offers a simplified way to identify circulating autoantibodies compared with peptides. It consists of a customized array containing 21,000 unique proteins that are allowed to interact with the serum of patients with a given disease compared to normal volunteers and/or control diseases. The difference in intensity of fluorescence is utilized for calculating the probability that a given protein represents an antigen interacting with a specific serum antibody. The advantage of proteins over peptides is the simplicity of calculation since the fluorescence intensity for a given protein is single, whereas with peptides, there is the necessity to calculate the alignment of 14 peptides. In this case, an antibody is considered specific for a given disease if the fluorescence intensity is higher than the upper limit of the normal range obtained from the analysis of 20 normal sera. Also, in this case, multiple analyses for complex data such as Volcano plots and hierarchical cluster analysis with a heat map are utilized for practical statistical use. It is important to note that the interaction of proteins with potential antibodies ‘in vivo’ involves the 3D structure directly mimicking what happens in vivo. This is an important difference in comparison with the linear peptides described above since they are not 3D. One of the drawbacks of protein arrays is the cost that has limited their clinical application to the area of drug development. For clinical research, a possibility is to design specific arrays in selected areas of study that could significantly reduce the number of proteins and, consequently, the price of the approach. One example is glomerular pathologies which could be investigated by using arrays containing around 500–1000 proteins corresponding to the protein composition of the glomerulus. In the case of specific diseases of the slit diaphragm, the number may be reduced further.

### 2.8. Miscellaneous Techniques

MS is the elective technique for the analysis of plasma proteins. It is a versatile technique that may be utilized in sequence with other approaches. However, one problem for MS is the highly dynamic range of protein concentrations in blood where few abundant proteins (corresponding to 80% of the total) cover the detection of the other low-concentration components. To overcome this challenge, the 20 most abundant blood proteins are usually depleted with specific antibodies immobilized on columns. Another possibility is to fractionate blood before MS. In both cases, the number of experiments for each analysis is notably increased.

Over the last decade, the Human Proteome Organization (HUPO) has developed guidelines for the proper identification of blood biomarkers, emphasizing the importance of quality standards and the selection of an adequate cohort of samples that ensure the statistical significance of the findings, the specificity of potential biomarkers, and their clinical application [19]. Current technological advances in blood proteomics have allowed the identification and quantification of approximately 1500 proteins in undepleted blood samples and over 10,000 unique proteins from fractionated blood samples [20].

In the study of circulating autoantibodies, MS should be combined with other approaches, such as the poll-down assay, to allow the identification and quantification of autoantibody antigens and their interactome (network of protein interactions).

## 3. Analysis of Cell-Producing Antibodies

The analysis of cell-producing specific antibodies adds crucial elements to the pathogenesis of any given autoimmune condition. Two techniques have been utilized for characterizing the origin of anti-PLA2R antibodies in MN.

The first technique consists of B-cell enzyme-linked immunoabsorbent FluoroSpot assays seeded with ‘ex vivo’ mononuclear cells, in which IgG antibody-secreting cells are detected with anti-human IgG conjugated with alkaline phosphatase, and PLA2R-specific IgG antibody-secreting cells are detected with fluorescent dye-labeled multimerized PLA2R monomers [21,22]. This technique enables quantification of the number of plasmablasts secreting PLA2R-targeted IgG over the global IgG-secreting plasma cell repertoire.

The second technology is based on Phage immunoprecipitation sequencing (PhIP-Seq) analysis, which involves a synthetic library of peptides or protein fragments of 40–90 amino acids on bacteriophage T7. This library is amplified and cloned in the T7 phage system and incubated with sera. The phage bound by serum IgG is precipitated with Protein A/G-coated magnetic beads. The fragment displayed (phenotype) is linked with the fragment encoded within the genome of each phage (genotype), enabling next-generation sequencing of precipitated phages and providing insight into serum antigen specificity. The affordability of PhIP-seq technology has led to its increasing use in autoimmune diseases, including type 1 diabetes, multiple sclerosis, rheumatoid arthritis [23], autoimmune encephalitis [24], and, most recently, post-COVID-19 multi-inflammatory syndrome in children [25].

## 4. Applications in Glomerular Autoimmunity

### 4.1. Goodpasture Syndrome

The discovery of renal autoantibodies started with Goodpasture syndrome, an autoimmune condition characterized by rapid evolution to end-stage renal failure and lung hemorrhages. Immunofluorescence studies indicated a thin linear deposition of autoantibodies along GBM, which suggested affinity for major structural components of the membrane such as collagen IV. The characterization of target epitopes of anti-GBM antibodies was a complex achievement that took several years. Early studies showed the reactivity of antibodies with the insoluble part of GBM and formed the basis for the characterization of two major target sites in the noncollageneous domain of the α3 and α5 chains of collagen IV (α3(IV)NC1) [26,27,28,29,30] and α5(IV)NC1 [31]. Two conformational epitopes, EA and EB, were recognized for each epitope and interact with many but not all circulating antibodies in Goodpasture syndrome [32,33]. Other noncollagenous targets have been successively recognized. One is laminin-512, which is prevalent in patients presenting pulmonary hemorrhage with hemoptysis [34], and the second is entactin, which forms bridges between collagen IV and laminin [35]. The discovery of different targets for anti-GBM antibodies has highlighted the heterogeneity of the disease. The bulk of the results deriving from decades of research have led tp the development of specific assays for a rapid and correct diagnosis of Goodpasture syndrome based on ELISA.

The passage from target antigen discovery and mechanisms of the disease is not yet completed. T-cell epitope mapping utilizing cells from patients or animals with experimental models of the Goodpasture syndrome is in progress and a mechanism linked with HLA functions is under investigation. HLA-DR15 confers an increased disease susceptibility while HLA-DR1 has a protective effect, suggesting that HLA polymorphisms result in structurally important differences in epitope HLA presentation to T cells [36,37]. The objective is to produce peptides that block the mechanism of HLA-DR1 presentation of GBM autoantigens to reactive T cells.

Finally, molecular mimicry may intervene in the pathogenesis of Goodpasture syndrome. It generically refers to immunological cross-reactivity between host antigens with bacterial antigens based on structural similarities [38]. In Goodpasture syndrome, mimicry of α3(IV)NC1 with *Actinomyces* may provide a stimulus to overcome immunotolerance in case of infections that are known to occur in the majority of patients at the disease onset [39,40].

### 4.2. Membranous Nephropathy

MN is the renal pathology that has shown more progress in the last few years. It is a primary autoimmune disease caused by circulating autoantibodies which have, in most cases, the kidney as a unique target. The discovery of the major two antigens of MN (PLA2R1 and THSD7A) was achieved by utilizing microdissection of glomeruli and two-dimensional electrophoresis in nonreducing conditions associated with mass spectrometry. Circulating anti-PLA2R1 antibodies [1] have been found in 65–70% of patients with MN and the levels have been correlated with the outcome of proteinuria and renal function after 12 months of follow-up. Further correlations with response to therapies have been described with circulating antl-PLA2R1 targeting specific epitopes of the protein, i.e., anti-CysRC1C7 [41,42,43,44]. Anti-THSD7A antibodies have been detected in a minority of cases (2–5%) [2,5]. A study carried out by Cantarelli et al. [21] recently attempted to characterize the cell origin of anti-PLA2R1 antibodies and the epitope recognized in the protein by antibodies by either the FluoroSpot assay or Phage immunoprecipitation sequencing. They did not find significant differences between MN patients, CKD patients, and healthy controls [21].

In the last 4 years, several other antibodies have been discovered using partial proteolysis and immunoprecipitation of frozen renal fragments [45]. The list of new antibodies includes anti-NELL1 [8], anti-SEMA3B [9], anti-PCDH7 [10], anti-HTRA1 [12], anti-NCAM1 [15], anti-FAT1 [46], anti-NetrinG1 [13], anticontactin1 [14], anti-NCAM1 [15], and anti-TGFBR3 [16]. Anti-EXT1 and anti-EXT2 [7] were found in a subset of patients with MN secondary to SLE. Knowing the antibody specificity can also help with following disease activity and may indicate potential associations such as cancer and intoxication.

In parallel with the above antibodies that recognize proteins specific to the kidney, another type of antibody detected in MN is anti-SOD2 [3], an intracellular detoxifying enzyme which is upregulated and externalized following autoimmune cell injury [47,48]. In the presence of anti-SOD2 antibodies, the anti-oxidative efficacy of SOD2 may be reduced, causing the block of protective functions of this enzyme. This would represent a negative and decisive event, leading to irreversible renal damage [5]. In the unique large study [5], considering a cohort of 230 patients with MN in which anti-SOD2 antibody serum levels were determined in parallel with the two major membrane-targeted autoantibodies, i.e., anti-PLA2R1 with anti-PLA2R1 epitopes and anti-THSD7A, anti-SOD2 antibodies emerged as the major factor associated with poor response to drugs and evolution to chronic renal failure.

Recently, Bruschi and colleagues [18] utilized peptide arrays to identify new circulating antibodies in MN. Several proteins representing potential new antigens were identified. Molecules recognize formin-like-1 (FMNL1), a protein endowed in the macrophage podosomes, that is implicated in macrophage movements. This finding points to the occurrence of a new kind of autoimmunity in MN that may modify the recovery phase of the pathology and have a role in the long-term outcome of the disease. As in other glomerular pathologies, the number of macrophages infiltrating the kidney increases proportionally with the severity of lesions, suggesting that macrophages have a role in determining progression.

Circulating antibodies in MN sera recognized a further eight proteins that represent further potential antigens. Their validation is in progress.

### 4.3. Lupus Nephritis

LN is the most frequent complication of SLE (occurring in 50% of all patients) [49] and has important clinical consequences for the severity of renal lesions and the frequent refractory to common treatments. It is an autoimmune glomerulonephritis of uncertain pathogenesis but with many candidate autoantibodies considered as potential drivers of glomerular lesions. Studies utilizing glomerular microdissection and proteomics have played a key role in furnishing evidence in favor of one or another candidate and the possibility is that many of them participate in different phases and support the evolving concept of a multifactorial origin. For some time, circulating anti-dsDNA autoantibodies have represented a reliable marker of SLE and SLE activity and have also been proposed by some authors as a clinical biomarker of LN. Association studies between anti-dsDNA levels and LN led, however, to inconclusive results in terms of sensitivity (range of positivity from 27 to 100%) and specificity (range from 13 to 89%), suggesting that anti-dsDNA has a limited value when making distinctions between SLE patients with and without nephritis [50,51]. Studies performed in a large series of Chinese patients with SLE with and without LN reported a significantly positive percent of anti-dsDNA in LN vs. SLE patients (63.3% of vs. 47.9%) that underlies the possibility of differences between Caucasians and Asians. Bruschi et al. [52,53] studied two large series of patients with SLE (n 561) and LN (n 481) and reported that anti-dsDNA (Farr test and ELISA) was indiscriminately high in both groups. The same authors described, in the same cohorts of patients, several other autoantibodies with specificity for SLE and LN; the predominant ones were anti-ENO1, anti-Histone2, and anti-ANXA1. An important aspect of circulating and renal autoantibodies in SLE and LN is that IgG2 is the prevalent isotype [54]. Further studies are necessary to further consider these new antibodies in the pathogenesis of LN and consolidate their potentialities as biomarkers of clinical outcome.

A customized peptide chip dedicated to LN has now been produced and has been utilized to characterize potential new antigens in SLE and LN. Initial results are of interest and require further confirmation utilizing ELISAs (Mn submitted).

### 4.4. Minimal Change Nephropathy

Circulating anti-nephrin antibodies have been only recently recognized in patients with idiopathic nephrotic syndrome (MCD/FSGS), a condition characterized by fusion of podocytes and, for many years, reputed to have a T-cell origin. The first ELISAs for anti-nephrin determination utilized the full-length extracellular domain of the protein that contains 6 IgG-like domains common to other proteins [55]. The results of a diffuse positivity in sera of MCD/FSGS patients were not reproduced in other laboratories. A re-evaluation of the specificity of the full-length nephrin and of single epitopes is now underway and the antigens utilized in ELISAs may change. A key problem of the anti-nephrin assay is that a few isoforms of the protein are expressed also in lymph nodes and the brain, and there is a need to better characterize the epitopes of renal isoforms interacting with circulating antibodies that are specific to the kidney. Recently, a two-step procedure with immunoprecipitation of IgG with Protein A followed by ELISA was shown to be more specific for the renal isotype [56]. Overall, the results indicate that circulating anti-nephrin is a characteristic of the less severe forms of MCD.

As described in the dedicated section, the immunoprecipitation technology is evolving and should lead to obtaining a more specific view of anti-nephrin antibody levels in MCD/FSGS. Magnetic beads and Western blot analysis of nephrin should furnish a more reliable view of this important topic. The confirmation of the existence of anti-nephrin antibodies and of their pathogenic role was obtained by analyzing renal tissue with advanced microscopy technologies (STED), in which case IgG and nephrin merge has been recognized in some MCD patients with less severe prognoses. The detailed inspection of many renal bioptic samples of patients with MCD using STED has also led to the conclusion that IgG binds more antigens than nephrin alone in the slit diaphragm, suggesting more research should be dedicated to this important area of nephrology. The same co-localization of nephrin with the punctate IgG was observed by confocal microscopy [17,57] in a cohort of patients.

An indirect confirmation of the autoimmune origin of MCD/FSGS may derive from the observation that anti-CD20 monoclonal antibodies are efficacious to induce prolonged remission of proteinuria [58,59,60] in the less evolute stages of the disease. It is clear that this is only a hypothetical conclusion since anti-CD20 antibodies may act also on other cells in the blood or may directly bind another receptor such as B7-1 in glomerli [61].

Last but not least, congenital nephrotic syndrome of the Finnish type, which is determined by mutations of nephrin, presents characteristics very similar to nongenetic idiopathic nephrotic syndrome (e.g., fusion of podocytes, loss of negative charges, etc.) [62]. The existence of circulating anti-nephrin antibodies has already been recognized after renal transplantation in patients carrying truncating mutations of the nephrin gene [63]. In this case, the normal nephrin of the renal graft is recognized as non-self by carriers of a truncated protein who develop, as a consequence, more antibodies, supporting the rationality of the model.

## 5. Conclusions

Technology innovation is playing a critical role in the discovery of autoantibodies in several autoimmune conditions and has fundamental importance for renal diseases. The implementation of mass spectrometry in laboratories for translational research has greatly contributed to this advancement. In renal pathology, mass spectrometry has been utilized for tissue analysis after microdissection of glomeruli or directly with immunoprecipitates derived from renal biopsies. This practice has contributed to the characterization of several new antibodies in autoimmune pathologies such as MN and lupus nephritis. Arrays utilizing peptides and proteins are now available and may be increasingly applied for the analysis of circulating antibodies. In spite of the limitation of costs, these new technologies probably represent the new frontier of translational research. Immunoprecipitation with Western blot analysis of nephrin and the demonstration of the existence of circulating anti-nephrin antibodies is changing the interpretation of renal conditions that were previously considered with minimal lesions (MCD) and should now be considered as ‘autoimmune diseases’. The strong proteinuric effect that the weak co-deposition of IgG with nephrin has in MCD completely modifies the sense of renal pathology. The technology evolution in microscopy has represented a key factor in this change. This is a topic in rapid evolution based on the commercial availability of STED. The new view on renal pathology that the use of STED has produced suggests that a more diffuse use would greatly impact this area.

Overall, the extension of new technologies of analysis from research to clinical practice is modifying the approach that is now required to reach a correct diagnosis. The most evident changes are in MN and MCD. In MN, the definition of the antibody responsible for the disease is fundamental in defining potential associations such as cancer or intoxication and, also, in determining the correct therapies or eliminating toxic substances. In MCD, the evolution of anti-nephrin antibodies, both circulating and in renal tissue, is adding some evidence to the pathogenesis of the disease and necessary modified therapies. Some questions are not resolved yet and require further research: one is related to the key point of pathogenesis (is anti-nephrin causative of nephrotic syndrome or is it an epiphenomenon?); the other is a matter of clinical interest (which type of nephrotic syndrome is more intensely associated with high anti-nephrin levels?). Finally, could circulating anti-nephrin levels be utilized for monitoring responses to drugs? The same questions apply to anti-nephrin antibody deposits within podocytes.

The necessity to address the points above for a correct clinical approach would require a substantial change in the organization of nephrology units, which will be obliged to increment the technology potential of their referent laboratories. Alternatively, the nephrology circuits should consider the constitution of collaborative units among different hospitals, which would represent an innovative approach to their organization.

## Abbreviations

Membranous nephropathy (MN); systemic lupus erythematosus (SLE); lupus nephritis (LN).
